# Impact of *APOE*, *Klotho,* and sex on cognitive decline with aging

**DOI:** 10.1073/pnas.2416042122

**Published:** 2025-02-04

**Authors:** Kengo Shibata, Cheng Chen, Xin You Tai, Sanjay G. Manohar, Masud Husain

**Affiliations:** ^a^Nuffield Department of Clinical Neurosciences, University of Oxford, Oxford OX3 9DU, United Kingdom; ^b^Department of Experimental Psychology, University of Oxford, Oxford OX2 6GG, United Kingdom; ^c^Division of Clinical Neurology, John Radcliffe Hospital, Oxford University Hospitals Trust, Oxford OX3 9DU, United Kingdom

**Keywords:** aging, antagonistic pleiotropy, apolipoprotein ε (*apoe*), cognition, *klotho*

## Abstract

The effects of apolipoprotein E (*APOE*) and *Klotho* genes, both implicated in aging, on human cognition as a function of sex and age are yet to be definitively established. Here, we showed in the largest cohort studied to date (*N* = 320,861) that *APOE* homozygous ε4 carriers had a greater decline in cognition with aging compared to ε3 carriers (ε3/ε4 and ε3/ε3) as well as smaller hippocampi and amygdala (*N* = 29,510). Critically, sex and age differentially affected the decline in cognition. Younger (40 to 50 y) female homozygous ε4 carriers showed a cognitive advantage over female ε3 carriers, but this advantage was not present in males. By contrast, *Klotho-VS* heterozygosity did not affect cognition or brain volume, regardless of *APOE* genotype, sex, or age. These cognitive trajectories with aging demonstrate clear sex-dependent antagonistic pleiotropy effects of *APOE* ε4, but no effects of *Klotho* genotype on cognition and brain volume.

## Significance

The influence of apolipoprotein E (*APOE*) and *Klotho* genes on cognitive health has been widely debated, with studies reporting both protective and detrimental effects. This study, using the largest population-based cohort to date, resolves much of this uncertainty. We demonstrate that carrying two copies of *APOE* ε4 is associated with accelerated cognitive decline with aging. However, female carriers of this gene exhibited improved cognition at younger ages. Contrary to our hypothesis, no protective effects of the *Klotho* gene were observed. These findings provide new insights into genetic risk stratification and guide therapeutic approaches for cognitive decline.

Several lines of evidence have suggested that genotype can influence the risk of cognitive decline and ultimately dementia (1, 2). However, the relative contribution of genetic and environmental risk factors on brain health continues to be vigorously debated (3, 4). Of all genes studied to date, the ε4 variant of the apolipoprotein E (*APOE*) gene has been most strongly identified as a significant risk factor for developing sporadic Alzheimer’s disease (AD) (1, 5–7). Even healthy carriers of the *APOE* ε4 gene have been reported to experience accelerated cognitive decline compared to noncarriers (8–10), along with notable structural and functional brain alterations (11–13) and localized effects on the hippocampus (14–17). As these effects are observed in asymptomatic individuals, before the onset of either mild cognitive impairment (MCI) or AD, genotyping provides a potentially powerful means of stratifying which individuals are more likely to develop dementia (18).

However, the literature on how *APOE ε4* influences the progression and manifestation of cognitive decline over the life span in healthy individuals remains mixed. Crucially, the directionality of the effects caused by carrying *APOE* ε4 on the brain has been perplexing, especially in younger individuals. Several studies have reported a cognitive advantage of carrying *APOE* ε4 at earlier ages (19–22). These findings suggest that the *APOE* ε4 variant may confer an advantage in early adulthood, but this comes at the cost of a higher rate of cognitive decline and increased risk of dementia in later life. This pattern aligns with the concept of *antagonistic pleiotropy*, where a gene that provides benefits in early life may lead to disadvantages with aging (23, 24). This early life benefit of *APOE* ε4 might account for its evolutionary persistence.

In addition to the significant impact of the *APOE* genotype on mediating cognitive decline, studies have also emphasized the potentially crucial role of an individual’s sex (25). Females compared to male carriers of the ε4 allele are reported to be at a higher risk of developing AD (26–28) and experience faster rates of memory decline in MCI (29). A memory advantage in male *APOE* ε4 carriers in midlife (40 to 51 y old) (30) and a cognitive disadvantage in female *APOE* ε4 carriers (50 to 80 y old) (31) has also been reported. Therefore, the strong interaction between sex and age in cognitive resilience and decline highlights the need to examine how sex uniquely modulates the impact of the *APOE* ε4 allele. However, the interplay between *APOE* genotype, age, and sex in influencing cognition remains to be definitively established (32).

The *Klotho* genotype is another genetic factor, alongside *APOE*, that is believed to influence cognitive function. The Klotho protein has been linked to increased lifespan when overexpressed in mice (33), while mice lacking this protein lived for shorter periods (34). Building on these seminal findings, human studies have explored *Klotho*’s potential impact on brain health. Carrying one, but not two copies of the KL-VS haplotype, i.e., *Klotho-VS* heterozygosity (*Klotho* VS/FC) has been identified as a genetic factor with a potentially protective effect, increasing longevity (35–37) and crucially also improving cognition (38, 39). *Klotho* VS/FC has also been associated with larger volumes of the right dorsolateral prefrontal cortex, and corresponding enhanced executive function, compared to *Klotho* homozygotes (40).

Mechanistically, *Klotho-VS* heterozygosity has been associated with an increase in serum Klotho protein levels (41) which has recently been shown to correlate with cognitive scores (42, 43). Critically, it has also been reported to be protective against amyloid deposition caused by *APOE* ε44 (44, 45). These *Klotho*-mediated genetic effects motivate the investigation into treatments targeting the *Klotho* pathway, especially in those with higher risk of developing AD. However, the effects of *Klotho* VS/FC are still under considerable debate (46–48). Conflicting findings have been reported, with one study indicating that *Klotho* VS/FC does not provide a cognitive advantage (49) and another showing shorter, not longer, survival in heterozygous carriers (48). Any benefits conferred by *Klotho* genotype may potentially be age or sex-dependent and therefore warrant further investigation in larger sample sizes.

Here, we aimed to investigate the polygenic effects of *APOE* and *Klotho* on cognition and brain structures across aging, utilizing an extensive dataset from the UK Biobank—the largest sample studied to date (50). Studying individuals before disease onset allows the assessment of risk factors in a healthy population to capture early signs of cognitive changes in a preclinical state. We established a composite behavioral score derived from a battery of cognitive tasks. In addition, structural MRI was used to extract gray matter volumes of predefined brain regions as a metric for brain integrity. We aimed to separately assess the role of each gene variant across age and sex on a composite score of cognition and gray matter volumes. The large sample size afforded by the UK Biobank allowed us to establish 1) the ages at which any effects of *APOE* and *Klotho* on cognition or brain structure appear 2) whether any of these effects are sex-dependent and 3) the interaction between these two genes. We hypothesized that *APOE* homozygous ε4 carriers would exhibit more rapid cognitive decline and yield neuroanatomical changes with aging, while individuals with *Klotho*-VS heterozygosity would exhibit protective effects against such decline (51, 52). By investigating these effects, we aimed to contribute to the current understanding of genetic predispositions to neurodegeneration in healthy individuals across sex and aging.

## Results

### *APOE*’s Effect on Cognition Is Age and Sex Dependent.

**Fig. 1.**
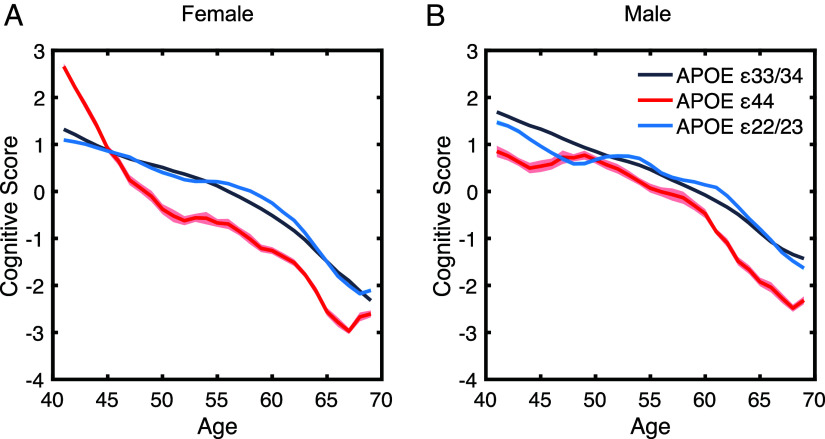
The influence of *APOE*, sex, and age on cognitive performance. Panel (*A*) (female) comparison of cognitive composite scores among *APOE* ε33/34 (gray), ε44 (red), and ε22/23 (blue) genotypes revealed genotype and age-dependent effects on cognition. Female ε44 carriers demonstrated a cognitive advantage over ε33/34 carriers until age 45, followed by a steeper decline. In ε22/23 carriers, compared to ε33/34, a modest cognitive improvement was observed between the ages of 55 and 65. Panel (*B*) (male) cognitive composite scores for males indicated that *APOE* ε33/34 consistently outperformed ε44 across all age groups. ε22/23 carriers exhibit a slight cognitive improvement during middle age. Cognitive scores are represented using a 3-y moving average with individual’s ages rounded to the nearest integer. The shaded area represents SEM for each age group.

**Fig. 2.**
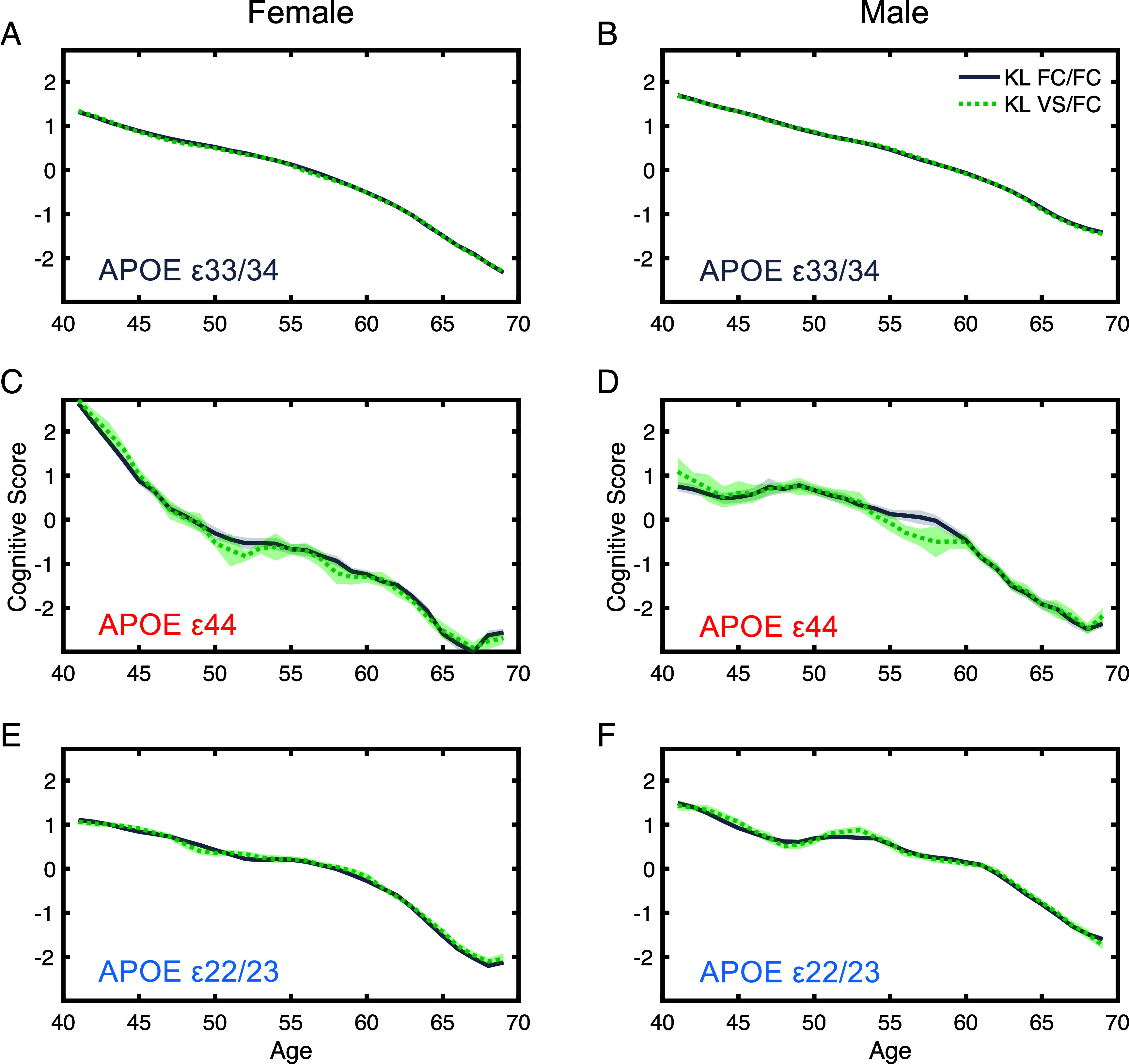
The influence of *Klotho*, sex, and age on cognitive performance. Panels (*A*–*F*) (*Left* Panels Female, *Right* Panels Male): Comparison of cognitive scores between *Klotho* genotypes (*Klotho* FC/FC vs. FC/VS) showed minimal differences in cognition, regardless of *APOE* genotype. Contrary to expectations, the anticipated cognitive protection in *APOE* ε44 carriers by the *Klotho* VS/FC genotype was not evident, as shown in panels (*C* and *D*). Cognitive scores are represented using a three-year moving average with individual’s ages rounded to the nearest integer. The shaded area represents the SEM for each age group.

To study the effect of *APOE* and *Klotho* genotype on the cognitive functions of healthy adults, a composite score was derived from nine cognitive tasks of 312,524 participants aged 40 to 70 by applying principal component analysis (PCA) and extracting the first principal component (*Methods* and Table 1). How this composite score of cognition varied with *APOE* genotype (ε33/34, ε44, ε22/23), *Klotho* genotype (FC/FC, VS/FC), sex (female, male), and age was assessed using multiple linear regression. This gave a factorial 3 × 2 × 2 × age design, with effects visualized in Figs. 1 and 2. Each main effect and interaction term without the age predictor reflected an effect at the baseline age of 40.

**Table 1.** Cognitive tasks of the UK Biobank

Reaction time task: This task measured reaction time to visual stimuli using a modified Go/No-Go format. The mean time taken to respond to matching pairs was used as the measure of reaction time.
Trail-making task: Trail-making assessed executive function. Trail A required participants to click on numbers in ascending order. Trail B required participants to click on numbers and letters in an alternating and ascending pattern (e.g., 1-A-2-B-3-C). The difference in completion time between Trail A and B was used as an index of executive function.
Pairs matching task: This task assessed visual memory. Participants memorized the position of as many matching pairs of cards arranged randomly on a grid. The cards were then turned face down and participants were asked to select as many pairs as possible in the fewest tries. The number of incorrect matches until all matches were made was used as the outcome measure.
Fluid intelligence task: This task measured IQ by probing verbal and numerical reasoning. Participants answered 12 multiple-choice questions (between 3 and 5 choices) that examined logic and reasoning ability. We used the fluid intelligence score to index their performance.
Paired-associative task: This task measured verbal declarative memory. Participants memorized word pairs displayed for 30 s. After a delay (Matrix Pattern Completion Task) one word of the pair was presented and the corresponding pair had to be selected from a choice of four options. The outcome variable was the number of correctly answered pairs.
Tower task: This task tested planning abilities. Participants were shown 2 displays of 3 pegs and 3 different colored hoops placed on the pegs. Participants counted how many moves it would take to go from one configuration of hoops to the other. The outcome variable was the number of items answered correctly in 3 min.
Symbol-digit substitution task: This task measured processing speed. Participants were presented with a key that paired symbols with numbers. Participants were shown a row of symbols and entered the associated number using a keypad. The score was the number of correct symbol-digit matches made in 60 s.
Matrix pattern completion task: This task tested nonverbal fluid reasoning. Participants were presented with a matrix design, with a missing piece in the lower right corner. Participants identified what the missing piece would look like from a list of options. The outcome variable was the number of items answered correctly in 3 min.
Numeric memory task: Short-term memory and working memory capacity was tested using a backward digit span task. Participants were simultaneously presented with a sequence of numbers and were asked to recall and repeat the numbers in reverse order. The outcome variable was the maximum number of digits correctly remembered in the reverse order.

Nine cognitive tasks from the UK Biobank were used to construct a composite cognitive score.

**Fig. 3.**
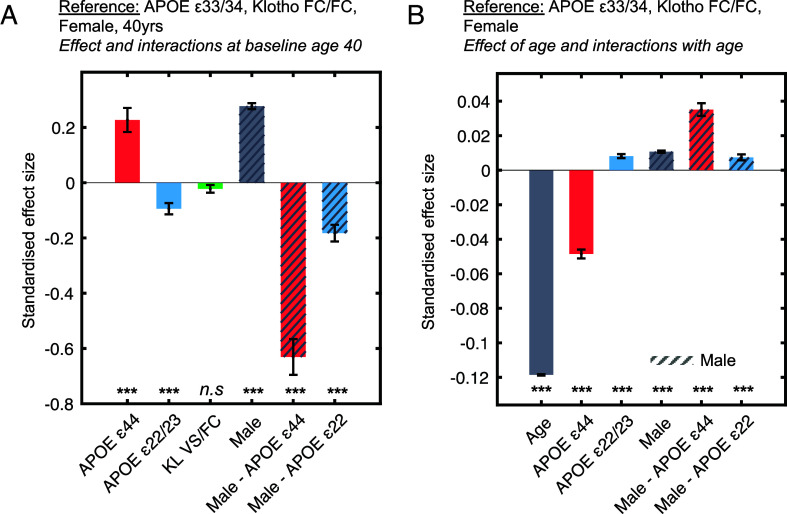
Multiple regression coefficients assessing the influence of *APOE*, *Klotho*, sex, and age on cognition. Panel (*A*) coefficients from the multiple regression, representing the main effects and the interaction between sex and *APOE* genotype. Effects of sex and genotype on cognitive performance are shown, where reference variables are *APOE* ε33/34, *Klotho* FC/FC, and female at baseline age of 40. Panel (*B*) coefficients from the multiple regression representing the effect of age on cognition, along with age interactions. Only statistically significant interactions are visualized. The cognitive composite score (units in SD) used is derived from the first principal component of cognitive tests from the UK Biobank. The units represent the estimated effect size for each predictor in terms of SD change per unit variable, representing the expected change in cognitive outcome (in SD units) for a one-unit increase in each predictor variable. Error bars represent the SEM. Significance levels are denoted as follows: *P < 0.05, **P < 0.01, ***P < 0.001.

At baseline age, carrying *APOE* ε44 positively affected cognition (β = 0.23, SE = 0.044, t = 5.21, *P* < 0.001) compared to ε33/34 (Fig. 1, panels *A* and *B*). By contrast, carrying *APOE* ε22/23 negatively affected cognition (β = −0.094, SE = 0.020, t = −4.65, *P* < 0.001). No significant main effect of *Klotho VS/FC* was observed (KL: β = −0.022, SE = 0.014, t = −1.58, *P* = 0.11, Fig. 2, panels *A*–*F*). *Klotho* status did not interact with sex (KL × sex: β = 0.030, SE = 0.021, t = 1.44, *P* = 0.15) nor *APOE* genotype (KL × *APOE* ε44: *Klotho* β = 0.052, SE = 0.081, t = 0.64, *P* = 0.52; KL × *APOE* ε22/23: β = −0.019, SE = 0.038, t = −0.49, *P* = 0.63) nor age (KL × age: β = 0.001, SE = 0.001, t = 0.79, *P* = 0.43). Therefore, the anticipated protective effects of *Klotho* VS/FC for *APOE* ε44 were not observed. The regression coefficients of these effects are plotted in Fig. 3.

Across all genotypes, at the baseline age of 40, males showed significantly better cognition than females (sex: β = 0.28, SE = 0.011, t = 25.34, *P* < 0.001). However, the interaction between *APOE* and sex revealed that at age 40, males had substantially lower cognitive performance in ε44 (ε44 × sex: β = −0.63, SE = 0.065, t = −9.71, *P* < 0.001) and ε22 (ε22 × sex: β = −0.18, SE = 0.030, t = −6.04, *P* < 0.001) compared to females of the same genotype. To reiterate, the directionality reported here only considers effects at the baseline age 40 (Fig. 3, panel *A*).

Age plays a pivotal role in cognitive decline, as demonstrated by the trajectories of cognitive performance across all panels (Figs. 1 and 2). Consistently, age significantly reduced cognitive performance (main effect of age: β = −0.12, SE = 0.00, t = −279.55, *P* < 0.001). The following analysis reports the predictors and their relationship with age (Fig. 3, panel *B*). The cognitive advantage associated with the *APOE* ε44 genotype observed at baseline diminished and reversed in older participants. Indeed, the expected negative influence of *APOE* ε44 on cognition increased with age (age × ε44: β = −0.049, SE = 0.003, t = −19.07, *P* < 0.001), underscoring an age-dependent and accelerated cognitive decline in *APOE* ε44 carriers. For carriers of *APOE* ε22/23, the slope of the cognitive change across age was less steep (slower) with aging compared to ε33/34 carriers (age × ε22: β = 0.008, SE = 0.001, t = 7.00, *P* < 0.001).

A significant age-by-sex interaction indicated that females experienced a faster overall decline in cognition with aging (age × sex: β = 0.011, SE = 0.001, t = 17.23, *P* < 0.001). Critically, this decline with age in *APOE* ε44 carriers was dependent on sex, with females being more strongly impacted by the gene (ε44 × sex × age: β = 0.035, SE = 0.004, t = 9.46, *P* < 0.001) (Visualized in SI Appendix, Fig. S1). The *APOE* ε22/23 effect with age, albeit weaker, was also modulated by sex, whereby the early female advantage declined faster with age (age × ε22/23: β = −0.18, SE = 0.030, t = −6.04, *P* < 0.001). The coefficients from the statistics are visualized in Fig. 3, panels *A* and *B* and summarized in Table 2.

**Table 2.** Regression model results for the effect of *APOE, Klotho*, sex, and age

Term	Estimate	SE	tStat	*P-*value
(Intercept)	1.657	0.007	225.59	<0.001 ***
*APOE* ε44	0.227	0.044	5.209	<0.001 ***
*APOE* ε22/23	−0.094	0.020	−4.654	<0.001 ***
*Klotho-VS/FC*	−0.022	0.014	−1.582	0.11356
sex	0.277	0.011	25.335	<0.001 ***
age	−0.119	0.000	−279.55	<0.001 ***
*APOE* ε44:*Klotho-VS/FC*	0.052	0.081	0.640	0.5223
*APOE* ε22/23:*Klotho-VS/FC*	−0.019	0.038	−0.488	0.62549
*APOE* ε44:sex	−0.631	0.065	−9.710	<0.001 ***
*APOE* ε22/23:sex	−0.183	0.030	−6.039	<0.001 ***
*Klotho-VS/FC*:sex	0.030	0.021	1.439	0.15007
*APOE* ε44:age	−0.049	0.003	−19.066	<0.001 ***
*APOE* ε22/23:age	0.008	0.001	7.001	<0.001 ***
*Klotho-VS/FC*:age	0.001	0.001	0.792	0.42854
sex:age	0.011	0.001	17.230	<0.001 ***
*APOE* ε44:*Klotho-VS/FC*:sex	0.000	0.121	0.003	0.99724
*APOE* ε22/23:*Klotho-VS/FC*:sex	0.015	0.057	0.263	0.79289
*APOE* ε44:*Klotho-VS/FC*:age	−0.006	0.005	−1.240	0.21511
*APOE* ε22/23:*Klotho-VS/FC*:age	0.004	0.002	1.950	0.051215
*APOE* ε44:sex:age	0.035	0.004	9.462	<0.001 ***
*APOE* ε22/23:sex:age	0.007	0.002	4.341	<0.001 ***
*Klotho-VS/FC*:sex:age	−0.001	0.001	−1.229	0.21895
*APOE* ε44:*Klotho-VS/FC*:sex:age	0.001	0.007	0.140	0.88869
*APOE* ε22/23:*Klotho-VS/FC*:sex:age	−0.002	0.003	−0.744	0.45668

Results of the multiple regression analysis, incorporating predictor: *APOE* (ε33/34, ε44, ε22/23), *Klotho* (FC/FC, FC/VS), sex, age, and all their possible interactions. Each row represents a predictor or interaction, with significant *P*-values highlighted in bold. The corresponding coefficients and SEs are visually represented in Fig. 3, Panels *A* and *B*.

### Age Determines the Influence of *APOE* Genotype.

To evaluate the interactions between *APOE* genotype and sex in different age categories, we partitioned the analysis into three age categories, 40 to 50, 50 to 60, and 60 to 70 y. This segmentation was chosen to divide the age ranges into equal intervals, facilitating the assessment of age-related trends in the effects of genes and sex on cognition. We excluded the *Klotho* variable from this follow-up analysis as it did not significantly predict cognition in the previously reported models. A separate age-partitioned analysis of the Klotho effect, presented in SI Appendix, Fig. S4, revealed no significant effect of Klotho in any age group.

**Fig. 4.**
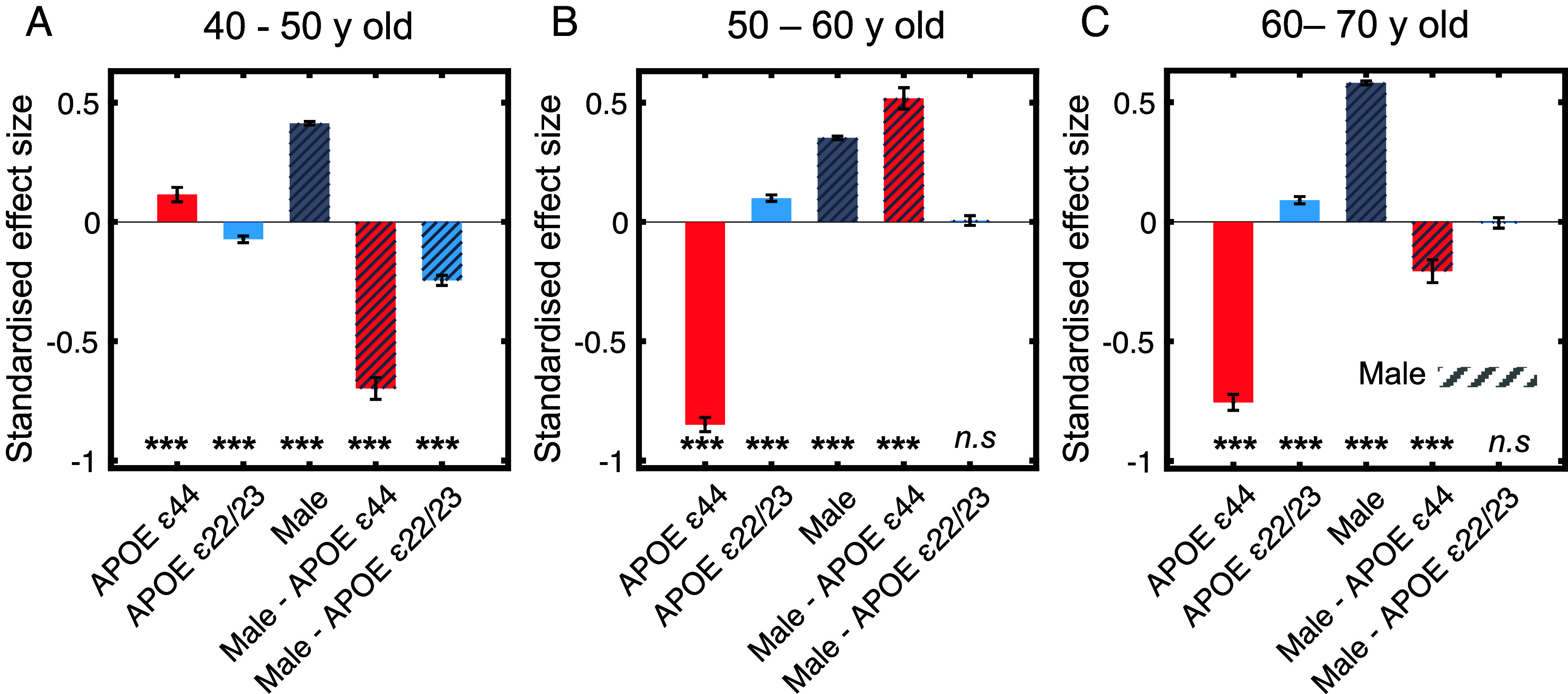
Effect of *APOE* and sex by age ranges. Panels (*A*–*C*) coefficient values for the main effects of *APOE* and sex, and their interaction, analyzed through subsampling across three age groups: 40 to 50, 50 to 60, and 60 to 70 y. The *Klotho* genotype was not significant in the previous regression and thus excluded from these analyses. The *y*-axis represents standardized effect size i.e., SD change in cognitive score per unit of the predictor variable. Error bars represent the SEM. Significance levels are indicated as *P < 0.05, **P < 0.01, ***P < 0.001.

The positive effect of *APOE* ε44 on cognition in the 40 to 50 age group (ε44: β = 0.12, SE = 0.030, t = 3.78, *P* < 0.001), reversed to a negative effect in the subsequent age brackets; 50 to 60 (ε44: β = −0.85, SE = 0.030, t = −28.46, *P* < 0.001) and 60 to 70 y (ε44: β = −0.76, SE = 0.033, t = −22.61, *P* < 0.001) as illustrated in Fig. 4, panels *A*–*C*. A similar pattern was observed for APOE ε22/23, which showed a negative effect on cognition in the 40 to 50 age range (ε22/23: β = −0.073, SE = 0.014, t = −5.14, *P* < 0.001), which transitions to positive effects in the 50 to 60 (ε22/23: β = 0.100, SE = 0.014, t = 7.35, *P* < 0.001) and 60 to 70 (ε22/23: β = 0.091, SE = 0.015, t = 6.03, *P* < 0.001) age groups. The effect of being homozygous for *APOE* ε4 is positive at younger ages and is only detrimental above 50 y of age.

Despite males generally displaying better cognition scores across all ages, a pronounced female advantage at ages 40 to 50 was shown by a significant interaction between sex and *APOE* ε44 (sex × ε44: β = −0.70, SE = 0.046, t = −15.27, *P* < 0.001) and *APOE* ε22/23 (sex × ε22/23: β = 0.52, SE = 0.045, t = 11.54, *P* < 0.001). However, this advantage was not apparent in the 50 to 60 age group (Male-*APOE* ε44 bar in dashed red). The interaction re-emerged in the 60 to 70-y-olds (sex × ε44: β = −0.21, SE = 0.048, t = −4.32, *P* < 0.001). Although female *APOE* ε22/23 carriers had a cognitive advantage between 40 and 50 (sex × ε22/23: β = −0.25, SE = 0.021, t = −11.47, *P* < 0.001), sex no longer affected this effect at older ages (50 to 60, sex × ε22/23: β = 0.006, SE = 0.020, t = 0.28, *P* = 0.78; 60 to 70, sex × ε22/23: β = −0.005, SE = 0.022, t = −0.23, *P* = 0.82) (Male—*APOE* ε22/23 bar in dashed purple). Detailed statistics for this model can be found in SI Appendix, Table S1. Overall, the positive effect of carrying *APOE* ε44 and ε22/23 was sex-dependent, with females showing a strong advantage between 40 and 50 in both genotypes.

### *APOE* e44 Associated with Medial Temporal Lobe Volume Loss But Increased Visual Cortex Volume.

**Fig. 5.**
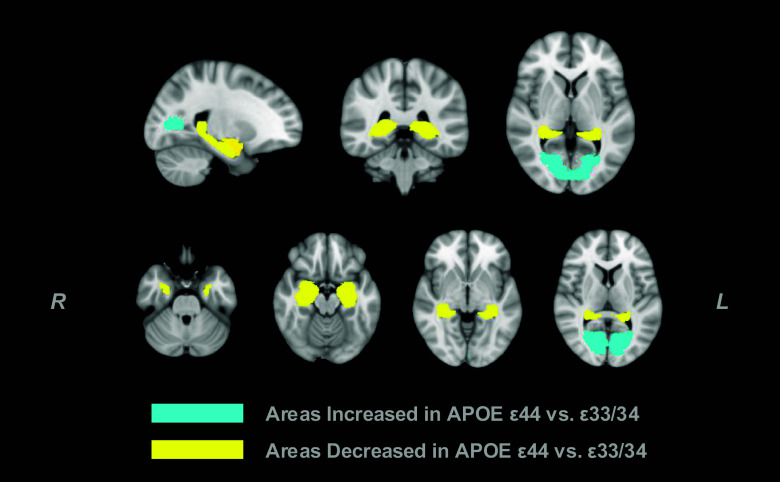
Brain regions modulated by *APOE* ε44 carriers compared to ε33/34 carriers Brain topography illustrates gray matter regions with significantly increased (light blue) or decreased (yellow) volumes. The top row shows a coronal, sagittal, and horizontal section of the brain using the MNI T2 template. The second row shows ventral to dorsal sections of the horizontal section. Region-specific decline by age is shown in Fig. 6.

We next investigated differences in brain volume across genotypes in both sexes. Multiple regression analysis with Bonferroni correction for 137 multiple comparisons yielded six gray matter regions that significantly differed between carriers of *APOE* ε33/34 and ε44 (Fig. 5). Regions with lower volumes in carriers of the ε44 variant were limited to the medial temporal lobe with medium effect sizes; left amygdala (f = 0.17), right amygdala (f = 0.20), left hippocampus (f = 0.10), right hippocampus (f = 0.095) (yellow). Albeit with a smaller effect size (f = 0.0029, 0.037 respectively), both left and right intracalcarine cortices were significantly increased in ε44 carriers (light blue).

**Fig. 6.**
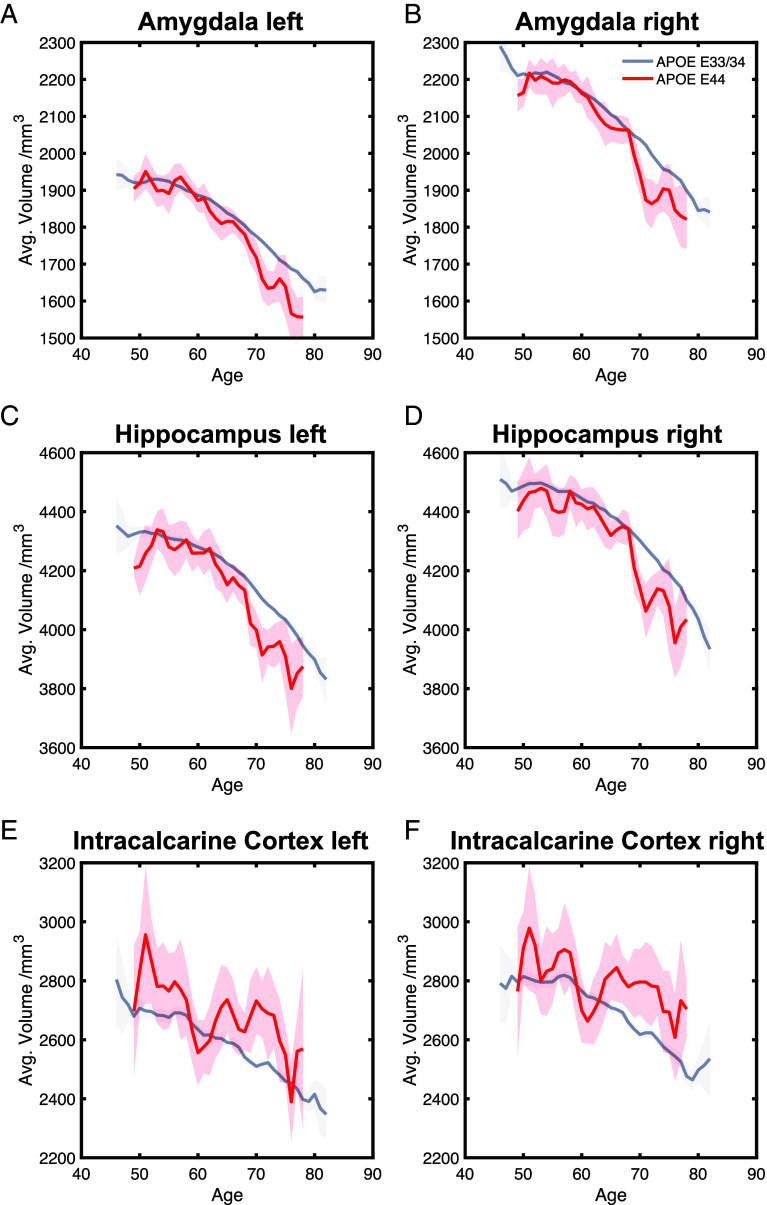
Effect of *APOE* ε44 on brain volumes. Panels (*A*–*D*) significantly reduced brain regions in individuals with the *APOE* ε44 compared to ε33/34. Panels (*E* and *F*) regions with significantly increased volumes associated with the *APOE* ε44 compared to ε33/34. These changes in brain volume are visualized using 3-y sliding averages. The shaded area represents the SEM for each age group.

A consistent and significant main effect of age was observed across all regions. The main effect of sex was significant for the left amygdala (β = 14.41, SE = 2.41, t = 5.97, *P* < 0.001) and left hippocampus (β = 3.36, SE = 0.90, t = 3.72, *P* < 0.001), where these regions were significantly larger in males. No other brain region was significantly modulated by sex. Importantly, there was no female advantage of brain volumes. Furthermore, no significant interaction effects between *APOE* genotype and sex were detected across the examined regions. ε22/23 did not have significantly modulated brain regions compared to ε33/34, nor did *Klotho* VS/FC carriers over *Klotho* FC/FC carriers (Fig. 6).

### Brain Volumes Correlate with Cognitive Scores.

**Fig. 7.**
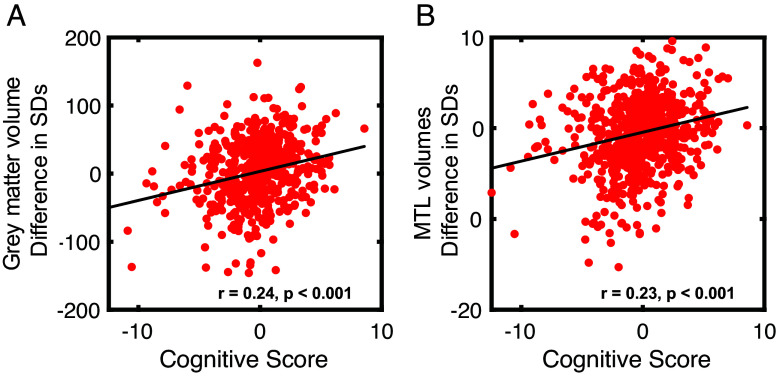
Correlation between brain volume and cognitive score in *APOE* ε44. Panel (*A*) total gray volumes (standardized units) are z-scored and plotted against an individual’s cognitive score. Panel (*B*) regions that were significantly affected by genotype (bilateral amygdala and hippocampus) plotted against an individual’s cognitive score. Correlations are reported as Pearson’s R.

Given that six brain regions were significantly different between *APOE* ε33/34 and ε44, we assessed whether the volumes of these regions correlated with cognitive scores. The composite cognitive score of carriers of *APOE* ε44 correlated with both total gray matter volume [r(659) = 0.24, *P* < 0.001] and the z-score sums of the regions that were significantly different [r(659) = 0.23, *P* < 0.001] (Fig. 7). We further examined whether only the enlarged regions (bilateral intracalcarine cortex) in *APOE* ε44 carriers compared to ε33/34 carriers would positively correlate with cognition. Although the directionality of the effect was as expected, we did not observe a significant correlation between enlarged areas and cognitive scores [r(659) = 0.07, *P* = 0.089].

To determine whether the effect of genotype (ε33/34 vs. ε44) on cognition was mediated by brain volumes, a mediation analysis was also conducted. This analysis examined whether the relationship between the *APOE* ε44 genotype and cognitive decline was mediated by brain volumes that were significantly reduced in *APOE* ε44 carriers compared to ε33/34 carriers. The results indicated that the ε44 genotype was significantly associated with cognitive decline, and this relationship was partially mediated by brain volumes. Specifically, the mediation analysis revealed that the association between the ε44 genotype and cognitive decline was attenuated when the indirect path involving brain volumes was included. This reduction was evidenced by a modest but significant indirect effect of β = 0.0172 (*P* = 0.007), as determined by the permutation test (*Methods*).

## Discussion

This study investigated the influence of apolipoprotein ε (*APOE*) and *Klotho* genotype, alongside the effects of age and sex on cognitive function and brain volume in the largest cohort of healthy individuals examined to date. The analysis revealed differential effects of *APOE* genotypes on cognition, as well as a notable absence of any effect of *Klotho VS/FC* heterozygosity. The interactions between *APOE* genotype, sex, and age, indicated a complex influence of sex and age on cognition. Most notably, the age-related cognitive trajectories showed an antagonistic pleiotropy effect, whereby the *APOE* ε44 gene associated with a later life disadvantage conferred an advantage at younger ages (23). However, this effect was limited to younger female carriers of the *APOE* ε44 variant, highlighting the importance of assessing *sex-specific* preclinical cognitive profiles for the early stages of dementia.

### The Deleterious Effect of ε44 on Cognition Emerges with Age.

*The APOE* ε44 genotype has been highly associated with the pathogenesis of AD (5, 6). Furthermore, *APOE* ε4 homozygosity has been suggested as a new genetic form of AD (7) and its risk in healthy individuals requires further investigation. Our study replicated the well-established decline in cognition of healthy *APOE* ε44 carriers compared to ε33/34 carriers (8–10). However, we found that in our cohort of healthy participants, ε44 carriers at the study baseline age (40 y) exhibited a cognitive advantage over ε33/34 carriers. This is consistent with *APOE* ε4’s association with enhanced cognitive performance in younger adults (19–22) and may be attributed to previously reported antagonistic pleiotropy effects (23). The strongest known genetic risk factor for AD is paradoxically associated with earlier advantages with resistance to infection (53), improved fitness during fetal development (54), enhanced fertility (53, 55) and here, higher cognitive abilities. Previous work has also indicated increased cortical thickness in ε4 carriers during their 40 s, followed by a more pronounced decline with aging (56). This may represent an initial structural advantage for ε4 carriers, possibly reflecting its effect on neurodevelopmental processes earlier in life, which subsequently transitions into accelerated cortical decline over time. This pattern suggests that effects associated with *APOE* ε4 may not be exclusively linked to neurodegenerative changes but could play a significant role in the broader neurodevelopmental trajectory (57, 58). Recognizing the potential role of these neurodevelopmental influences of *APOE* ε4 genotype may improve our understanding of the antagonistic pleiotropy mediated by this gene, which likely explains the persistence of this AD-risk-conferring gene in our genome.

### Antagonistic Pleiotropy of Cognition Is Sex-Specific.

Cognitive trajectories by age and sex revealed that female *APOE* ε44 carriers up to ~45 y of age displayed a cognitive advantage compared to female *APOE* ε33/34 carriers. However, this advantage was not observed in males. Furthermore, female ε44 outperformed ε44 males in cognition in this younger age range. The ε44 female advantage in cognitive performance diminished with age. Overall, males exhibiting higher cognitive abilities from mid-life onward, consistent with previous findings that males exhibit higher cognitive abilities at mid-life (30). This finding is also supported by studies showing that female ε44 carriers are more likely to go on to develop AD (28), have greater atrophy of the hippocampus (17) and are at a higher risk of AD pathology (59). Furthermore, these results are aligned with a meta-analysis assessing the link between *APOE* ε4 and sex differences, reporting that age differentially affected the risk of developing MCI and AD, with an increased risk of developing AD in women between 65 and 75 y of age (27). Therefore, age strongly impacts the sex-mediated decline in cognition, where we identify both an early-age advantage and a later-age decline. Further assessment of the mechanisms for age-related effects in females may be relevant to assess in terms of the recently characterized tau pathology in females (60–62). Given the impact of sex on brain health concerning AD, exploring sex-specific therapies might help to elucidate mechanisms underlying sex-related vulnerabilities across different ages (63). Further investigation in younger age groups is required to fully characterize the sex and genotype-specific effects across the lifespan.

### *APOE* ε44 Effects on Brain Structure.

The analyses performed here also revealed that carrying *APOE* ε44 was associated with smaller hippocampi and amygdala. These gray matter volume differences also correlated with the cognitive score and mediated the effect of ε44 on cognition, suggesting a potential structural basis for the cognitive decline observed in these individuals in later life. The small but significant correlations reflect the potential association between brain anatomy and cognition. These findings are aligned with studies showing that genetic predispositions, especially *APOE* ε44, can affect the hippocampus in healthy individuals (15, 64), even at the synaptic level, where hippocampal synaptic density was shown to be lower in cognitively unimpaired homozygous ε4 carriers (65). In this cohort, ε44 carriers also had enlarged bilateral intracalcarine cortices, consistent with a previous study on the same cohort reporting that the intracalcarine cortex correlated with the number of copies of ε44 alleles (66). While this dataset does not allow for causal inference, one potential explanation may be a compensatory recruitment process. For example, frontal activation in ε44 carriers has been discussed in relation to AD-related processes (23, 24, 67). This potentially protective or compensatory mechanism may contribute to the enlargement of the intracalcarine cortices in this genotype. Given its strong association with the visual cortex, this feature may also underlie the superior cognitive performance observed in ε44 carriers (22, 68), which is reported to last through aging in some visual domains (69). However, a correlation between bilateral intracalcarine cortex volume and cognition was not found. The analyses conducted here also showed an enlarged left amygdala and left hippocampus in males compared to females. These brain areas are important regions related to the neuropathological lesions of AD (70). No significant association of female early advantage could be identified in gray matter volumes, although previous studies have shown reduced functional connectivity to the hippocampus in female *APOE* ε4 carriers (16). Finally, no antagonistic pleiotropy effects were found in brain volumes, likely owing to the limited sample size that prevented equivalent analysis of age-related decline.

### *APOE* ε22/23 Effects.

The effects of the *APOE* ε2 variant were also assessed in comparison to the reference group of *APOE* ε33/34. The ε2 allele is associated with a lower risk of AD (71) and increased longevity (72). ε2 carriers are also more likely to live up to 100 (73), but the cognitive trajectory across aging has not been clearly defined. When comparing the effects of carriers of the ε22/23 genes, we found that ε2 carrier advantages became evident with aging. The cognitive decline with age was less steep compared to the reference ε33/34 carrier group. This highlights the potential protective effect of this allele, especially with age. Sex also modulated the effects whereby the positive effects of ε22/23 were higher in females at younger ages but changed to a male advantage with aging as was the case for ε44 carriers. Although some previous studies have reported increased cortical thickness (74) and larger hippocampal volume in healthy elderly ε2 carriers (75), reports are mixed with others showing no differences (76). Here, we also showed no difference in hippocampal volume.

### Absence of *Klotho* Effect.

While the benefits of *Klotho*-VS heterozygosity have been reported across species (33, 34, 36, 38), this study did not reveal any significant effects of *Klotho*-VS heterozygosity on cognitive function across aging. No effects on brain gray matter volumes were observed either. This contrasts with previous findings reporting improved memory and executive functions in *Klotho* heterozygotes, which correlated with a larger prefrontal cortex (38). Furthermore, *Klotho* VS/FC carriers did not provide neuroprotective effects to homozygous *APOE* ε4 carriers, contrary to prior findings (51, 52). This cohort study raises questions about whether the *Klotho* gene may have a counteracting effect on the degeneration induced by carrying *APOE* ε4.

One potential explanation for the mixed findings in the literature is the lack of a direct correlation between *Klotho* genotype and Klotho protein levels. Recent studies demonstrating the beneficial effects of Klotho secretion have directly evaluated Klotho levels through injection (74) or quantification of serum (42) and plasma (77) protein levels. For example, individuals with AD had lower Klotho concentrations in the cerebrospinal fluid compared to non-AD individuals (78). Klotho levels also correlate with neuropsychological test scores (79). Alongside transcriptional processes, Klotho secretion is highly modulated by environmental factors like chronic stress (80), which unlike socioeconomic status and education, is uncorrected for in this study. Therefore, *Klotho* genotype may not be a direct reflection of Klotho secretion. This is in agreement with a study reporting that Klotho protein levels, but not the *Klotho* genotype, were related with AD and amyloid and tau burden (81). The neurological outcomes appear more clearly mediated by Klotho protein levels rather than *Klotho* genotype, and therefore further effort into characterizing Klotho protein level is likely to be of increasing relevance for future studies.

Conflicting reports regarding the lack of longevity effects associated with *Klotho* VS/FC have raised questions about its clinical utility as a therapeutic target (79). There is also mixed evidence of the effects on cognition, which helps put the findings of this study into context. Genetically predicted levels of *Klotho*, as used in this study, were not associated with developing dementia in a large-scale observational study using neuroimaging (43). A similar conclusion was drawn in a population-based sample aged 55 to 87 (82). Furthermore, KL-VS genotype was associated with better cognitive function in younger individuals but not in older sample (83) and the unexpected KL-VS heterozygote survival and white matter volume disadvantages have also been reported in the Aberdeen Birth Cohort (48). To further characterize *Klotho* effects across the lifespan, studies have focused on cognition and brain structure in childhood and adolescence (84). Here, researchers found minimal evidence of KL-VS genotype affecting cognition and brain structure. However, interactions with genotype and age were apparent, whereby KL-VS heterozygotes showed better cognition before the age of 11, but worse in adolescence. Heterozygotes also had larger brains in early childhood. However, these findings could not be replicated in a separate sample, and some tasks were performed better in homozygous individuals. Last, in relation to *APOE* in this study, *Klotho* was not associated with amyloid beta and cognitive decline in individuals with *APOE* ε44 (47). This is in alignment with our results that KL-VS does not modify the decline in cognition mediated by *APOE* ε4 status. Overall, the positive effects of KL-VS have been called into question across various cohorts, and its neuroprotective properties require further investigation.

*Klotho* plays a critical role in calcium homeostasis and insulin signaling (33, 85). The mechanisms through which *Klotho* may confer its benefits remain an area of active research. These mechanisms could provide insight into the interplay between protein levels and genotype, as well as the age-dependent variation in physiological functions that may require higher levels of Klotho.

### Importance of Large Sample Sizes.

Understanding the progression of neurodegeneration requires assessing early changes preceding clinical diagnosis, a task facilitated by the UK Biobank data. The impact of genes has often been overshadowed by a focus on disease associations rather than risk factors in the healthy population. Here, we offer a perspective by investigating genetic risk factors, age, and sex in a large number of healthy participants. This cohort is the largest sample of *APOE* ε44 carriers to date, which allows us to investigate varying factors influencing brain and behavior. Furthermore, the characterization of trajectories over time is a critical aspect of the analysis, which has been overshadowed in previous investigations assessing the interactions of *Klotho* and *APOE* genes of aging (46). Inconsistencies in previous research regarding cognitive advantages among ε4 carriers may stem from limited statistical power, obscuring assessments of sex and age-related effects.

### Study Limitations.

Although the UK Biobank provides a valuable resource for studying cognitive impairment, several limitations warrant discussion. First, the data analyzed are cross-sectional. The large dataset of individuals of varying ages allowed the characterization of age-related decline in the composite cognitive score. However, longitudinal cognitive assessments, alongside the growing follow-up imaging data, are likely to provide even more comprehensive evaluations of cognitive trajectories. Second, our analysis reduced various cognitive tasks into a composite score using PCA, and therefore overlooked the variations across different cognitive domains. This analysis method was motivated by the lack of validated domain sensitivity of the tasks administered in the UK Biobank. While general cognition is important to study, specific cognitive domains should also be considered in a hypothesis-driven manner, as some domains of cognition may be differentially affected. For example, in the context of *APOE*, short-term and long-term retention has been shown to exhibit distinct effects and may warrant separate consideration (68). Improved cognitive testing which is sensitive to subtle cognitive deficits may facilitate this line of research. Third, as this cohort is made of healthy individuals, clinical conversion is not assessed, which may be critical to assess as not all cognitive decline in healthy is associated to pathological states. Last, the UK Biobank dataset is limited to healthy middle-aged and older adults. This restricts our ability to examine cognitive profiles and brain changes during earlier stages of adulthood. Particularly given the antagonistic pleiotropy effects characterized in female *APOE* ε44, how genotype affects cognition in early adulthood is of interest. Datasets such as the Adolescent Brain Cognitive Development Study (https://abcdstudy.org/), which specifically investigates early life focused on younger individuals may offer promising opportunities to disentangle the age-related interactions of genetic factors across the lifespan. Notwithstanding these limitations, our study’s broad age range and large sample size provide a strong foundation for future research on how to better stratify those at risk of developing dementia.

Education has been reported to be a significant factor in mental (86) and physical health (87). Previous studies investigating the same UK Biobank cohort have characterized how cognitive trajectories across aging are moderated by education (88, 89). In the present study, we controlled for educational attainment to isolate the effects of *APOE* and *Klotho* genotype and its interaction with sex and age on cognitive decline. While educational influences were not the primary focus of our analyses, how education across genotype and sex shape cognitive outcome is also of importance for future studies. This may further inform how sex-specific cognitive resilience patterns are mediated by nongenetic factors such as education.

## Conclusions

This study leveraged one of the most extensive genotyped cohorts of healthy individuals from the UK Biobank. The complex interplay between *APOE*, sex, and age, contributes to our understanding of how genetic effects on cognition are highly dependent on sex and age in both carriers of *APOE* ε4 and ε2. The female-specific antagonistic pleiotropy effects highlights the importance of assessing sex-dependent cognitive trajectories of dementia. Finally, despite the absence of significant effects from *Klotho*-VS heterozygosity on cognition, the mechanisms of *Klotho* on cognition still warrant investigation. Protective genetic factors that may modify the deleterious effects of *APOE* ε4 in later life remain a critical target for the development of therapies and diagnostics against aging and neurodegeneration.

## Methods

### Participants.

The participants in this study were drawn from the UK Biobank (https://ukbiobank.ac.uk), a population-based prospective cohort of healthy adults. 320,861 participants between the ages of 40 and 70 y were included in this study. Individuals with a history or current diagnoses of the following were excluded from the study: AD or Parkinson’s disease, stroke, brain/head injury, transient ischemic attack, subdural or subarachnoid hematoma; infection of the nervous system; brain abscess, hemorrhage, skull fracture, encephalitis, amyotrophic lateral sclerosis, meningitis, chronic neurological problem, multiple sclerosis, and epilepsy. Those with alcohol, opioid, and other dependencies were also removed from the analysis. Follow-up self-reported questionnaires and hospital records were used to assess inclusion in the study. Participants were genotyped during the data collection phase. Rarer variants of *APOE* (ε1) and *Klotho* [homozygous (VS/VS)] were not included in the analysis. Data were acquired from an initial baseline visit between 2006 and 2010, and the imaging visit occurred between 2014 and 2020. The imaging dataset is a small subset (N = 29,510) of those tested at the initial assessment. A general description of the demographics is provided in SI Appendix, Table S2. Informed consent was obtained from all UK Biobank participants. The study protocol was overseen by the UK Biobank Ethics Advisory Committee (www.ukbiobank.ac.uk/ethics). Ethical approval was granted by the North West Multi-center Research Ethics Committee (MREC), REC number 11/NW/038.

### Genetic Grouping.

The effects of carrying the *APOE* ε44 genotype were studied by comparing them to ε33 and ε34 carriers. For further exploratory analysis, we also compared ε2 carriers (ε22 and ε23) to the same reference (ε33 and ε34 carriers). The homozygous ε44 genotype was analyzed relative to the reference group, as it is widely recognized for exerting the most significant impact on cognitive function and brain changes and a potential genetic form of AD (7). Previously, the scarcity of homozygous carriers has limited this grouping; however, this limitation was overcome by the large sample size provided by the UK Biobank.

We assessed the dose-dependent effect of carrying an ε4 allele, by separating out the ε3/ε3 and ε3/ε4. Across both sexes, we found that ε3/ε3 carriers had higher cognitive scores compared to ε3/ε4 carriers, (coefficient = −0.015, SE = 0.0047, t = −3.27, *P* = 0.001), but the difference between ε3/ε4 and ε44 carriers were substantially greater (coefficient = 0.57, SE = 0.013, t = −44.85, *P* > 0.001, SI Appendix, Fig. S2). The slopes with age were significantly different in homozygous ε4 carriers compared to ε3/ε3 carriers (coefficient = −0.050, SE = 0.0021, t = −23.49, *P* < 0.001) but not when comparing heterozygous ε4 carriers with ε3/ε3 carriers (coefficient = −0.00025, SE = 0.00079, t = −0.32, *P* = 0 .75). We therefore combine ε3/ε3 and ε3/ε4 and assess the difference with ε44 carriers, with the greatest impact on cognition across aging.

We also assessed the differences in carrying one vs. two ε2 alleles on cognition. We found that ε2/ε3 carrier had marginally higher cognitive scores compared to ε3/ε3 (coefficient = 0.012, SE = 0.0061, t = 2.01, *P* = 0.044), but ε2/ε2 carriers did not (coefficient = −0.023, SE = 0.026, t = −0.91, *P* = 0.36). In addition, the effect of carrying *APOE* ε2 depended on age (ε2/ε3: coefficient = 0.0090, SE = 0.0010, t = −8.75, *P* < 0.001; ε2/ε2: coefficient = 0.014, SE = 0.0042, t = 3.33, *P* < 0.001), where in both cases ε3/ε3 outperformed ε2 carriers in early ages (ε2/ε3: coefficient = −0.098, SE = 0.018, t = −5.51, *P* < 0.001; ε2/ε2: coefficient = −0.21, SE = 0.073, t = −2.92, *P* = 0.0034). Both genotypes showed comparable trajectories with age (SI Appendix, Fig. S3) and have been grouped for the purpose of the main analysis.

For *Klotho*, the *Klotho*-VS heterozygous variant (FC/VS) was compared to the most common *Klotho* variant (FC/FC). 37,976 individuals had brain MRI data which was used to analyze differences in gray matter volume.

### Cognitive Testing.

Cognitive testing was performed via fully automated touchscreen tasks. We analyzed nine short cognitive tasks covering a range of cognitive domains, including processing speed, episodic memory, working memory, and reasoning (Table 1). High validity and test–retest reliability of these UK Biobank tasks have been reported previously (90).

### Behavioral Analysis.

Analyses were carried out on MATLAB R2021b. To examine the effect of genotype on cognitive functions, behavioral measures of each task were first z-scored. For tasks where lower values denoted superior performance (i.e., reaction time task, trail-making task, and pair-matching task), the values were adjusted so that negative z scores reflect below-average performance. Missing values were extrapolated using a matrix based on the age, sex, and *APOE* genotype of this participant. PCA was applied to these scores to reduce dimensions and establish a composite score of cognition. The 1st principal component was taken as the composite score of cognition for each individual. These scores were corrected for socioeconomic status using the Townsend deprivation index. As education is known to be a mediating factor in cognition, we have corrected the cognitive score for educational attainment. This aimed to better focus on the effects of genotype, sex, and age.

The composite score of cognition aimed to 1) reduce the number of comparisons across cognitive tasks, and 2) to focus on a general measure of cognition that might reflect cognitive functioning. Given the nature of the tasks (Table 1), the composite measure is likely to closely represent a variable reflecting executive functioning and working memory.

We investigated the factors affecting cognition using multiple linear regression, modeling cognition as a function of *APOE*, *Klotho*, sex, and age, and their interactions.

[1] $$\begin{array}{c} \text{Cognition}∼1+\text{APOE}+\text{Klotho}+\text{Age}+\text{Sex}\;+\left(\text{APOE}\times \text{Age}\right)\\ \;+\left(\text{Klotho}\times \text{Age}\right)+\left(\text{Sex}\times \text{Age}\right)+\left(\text{APOE}\times \text{Sex}\right)\\ \;+\left(\text{Klotho}\times \text{Sex}\right)+\left(\text{APOE}\times \text{Klotho}\times \text{Age}\right)\\ \;+\left(\text{APOE}\times \text{Klotho}\times \text{Sex}\right)+\left(\text{APOE}\times \text{Sex}\times \text{Age}\right)\\ \;+\left(\text{APOE}\times \text{Klotho}\times \text{Sex}\times \text{Age}\right). \end{array}$$

For the purpose of this analysis, the reference genotype was set to the most common grouping of variants of each of the genes studied; *APOE* ε33/34 and *Klotho* FC/FC. The reference sex was set as female. The minimum age was subtracted from all ages so that all main effects and interaction terms without the age predictor represent an effect at the minimum age in the data of 40 (*APOE* ε33/34: n = 3,361, *APOE* ε44: n = 129, *APOE* ε22/23: n = 513). This regression model explained 44.2% of the variance in cognitive scores (R-squared = 0.442), with a highly significant fit (F = 12,700, *P* < 0.001). We used the Akaike Information Criterion (AIC) and Bayesian Information Criterion (BIC) to confirm the superiority of this complex model over a model without interactions between predictors. As our primary focus was the effects of genotype and its relationship with age and sex, we opted for a model including various interaction terms. The full model had a lower AIC (AIC: 945,900) compared to the simple multiple regression model (AIC: 947,490) with no interactions and explained 42.2% of the variance compared to 41.9% variance. The model incorporating all interactions revealed a significant age effect, prompting further analysis of the directionality of *APOE* and sex across three age brackets: 40 to 50, 50 to 60, and 60 to 70 y. This segmentation equally divides the age ranges into three groups to assess whether the directionality of main effects changes depending on age. As *Klotho* did not influence the results, its effect was excluded from these subsequent age-binned regressions. Each age group was analyzed using the below regression model:

[2] Cognition∼1+APOE+Sex+(APOE×Sex).

In order to visualize the regression results, the coefficients of each significant independent variable were plotted in Figs. 3 and 4. The estimated effect size of each predictor is visualized in terms of SD change in cognitive score per unit of the predictor variable. This reveals the expected change in the composite cognitive score in SD, as the cognitive score is standardized with a mean of 0.

### Imaging.

MRI data were acquired on Siemens Skyra 3T scanner as part of the UK Biobank neuroimaging protocol, in a follow-up visit, on average around 10 y after the initial assessment. The imaging protocol was gradually implemented following the initial assessment, during which cognitive testing was conducted. 1 mm isotropic voxel high-resolution T1-weighted images were collected and preprocessed using a standard previously published pipeline implementing quality control (91). For volumetric analysis of T1-imaged structures, gradient distortion correction and registration onto MNI152 space were applied. FMRIB’s Automated Segmentation Tool was used to segment white matter, gray matter, and cerebrospinal fluid. We extracted 137 imaging-derived phenotypes as key gray matter structures using the Oxford-Harvard parcellation atlas. Volumes were corrected for years of education, socioeconomic status, head size, scan date, and table position using a general linear model. We examined the difference in gray matter volumes using a whole-brain approach across genotypes. We ran a multiple linear regression for each brain region and reported the brain regions that had a significant main effect of genotype. To correct for the multiple comparisons made across all brain regions, we applied Bonferroni correction with an adjusted alpha threshold of 0.05/137. Given the substantially smaller sample size and multiple regressions needed to be run for whole brain analysis, linear models which included interactions did not show any significant main effect or interaction effects. We therefore assessed the additive effects of genotype, sex, and age independently on gray matter volume using the following regressions. Regression models for ε44 and ε22/23 were assessed separately. Cohen’s f was used as a measure of effect size.

*APOE* ε33/34 vs. *APOE* ε44 and *APOE* ε33/34 vs. *APOE* ε22/23 separately:

[3] $$\mathrm{Gray}\;\mathrm{Matter}\;{\mathrm{Volume}}_{T}\;∼\;1\;+\;\mathrm{APOE}\;+\;\mathrm{Sex}\;+\;\mathrm{Age}.$$

T for every gray matter volume

*Klotho* FC/FC vs. *Klotho* VS/FC

[4] $$\mathrm{Gray}\;\mathrm{Matter}\;{\mathrm{Volume}}_{T}\;∼\;1\;+\;\mathrm{Klotho}\;+\;\mathrm{Sex}\;+\;\mathrm{Age}.$$

T for every gray matter volume

### Brain Behavior Correlations.

All brain volumes used for brain behavior correlation analysis were z-scored. A mediation analysis was conducted to examine whether the reduction in brain volumes mediates the relationship between the *APOE* ε44 genotype and cognitive decline. For the purpose of this hypothesis-driven analysis, participants with the *APOE* ε22/23 genotype were excluded, resulting in a binary *APOE* variable (ε44 vs. ε34/33). Volumes that were significantly reduced in carriers of *APOE* ε44 were z-scored and summed to create a composite measure of medial temporal lobe volume reduction. Logistic regression (*APOE* ~ Volume) and linear regressions (Cognitive Score ~ Volume and Cognitive Score ~ Volume + *APOE*) were performed to estimate the coefficients for the mediation model. The observed indirect effect (a * b) was calculated as the product of the coefficients from these models. A permutation test with 1,000 permutations was conducted to assess the significance of the indirect effect by permuting the *APOE* variable and recalculating the indirect effect for each permutation. The *P*-value was determined by comparing the observed indirect effect to the distribution of permuted indirect effects.

## Supplementary Material

Appendix 01 (PDF)

## Data Availability

UK Biobank data are openly accessible through www.ukbiobank.ac.uk (50).
