# Supplementary material for: Impact of APOE, Klotho, and sex on cognitive decline with aging
Source: Proc Natl Acad Sci U S A. 2025 Feb 4;122(6):e2416042122. doi: 10.1073/pnas.2416042122 (PMC11831164; doi:10.1073/pnas.2416042122)
Supplement: Supplementary file 1 — Appendix 01 (PDF) [file pnas.2416042122.sapp.pdf]

Supporting Information for

Impact of *APOE*, *Klotho* and sex on cognitive decline with aging

Kengo Shibata[1#], Cheng Chen[2], Xin You Tai[1,3], Sanjay G Manohar[1,2,3] & Masud Husain[1,2,3]

[1.] Nuffield Department of Clinical Neurosciences, University of Oxford, Oxford, UK
[2.] Department of Experimental Psychology, University of Oxford, UK
[3.] Division of Clinical Neurology, John Radcliffe Hospital, Oxford University Hospitals Trust, Oxford, United Kingdom

[#]Corresponding author
Kengo Shibata
Address: Nuffield Department of Clinical Neurosciences. Level 6, West Wing, John Radcliffe Hospital, University of Oxford, Oxford, OX3 9DU, United Kingdom
Email: kengo.shibata@ndcn.ox.ac.uk

This PDF file includes:

Supplemental Figures 1-4
Supplemental Tables 1-2

Impact of APOE, Klotho and sex on cognitive decline with aging
Supplemental Figures & Tables

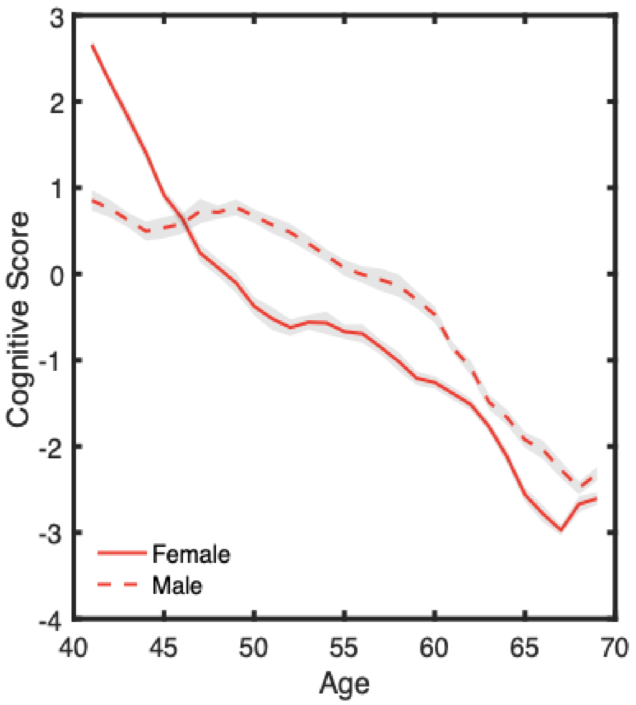

Supplemental Figure 1: Sex effects on cognition among *APOE* ε44 carriers
A cognitive advantage for females up to the age of 45, followed by a rapid decline compared to males if found. The cognitive composite score, used for visualisation and regression, is based on the first principal component of cognitive tests from the UK Biobank. Error bars represent the standard error of the mean (SEM).

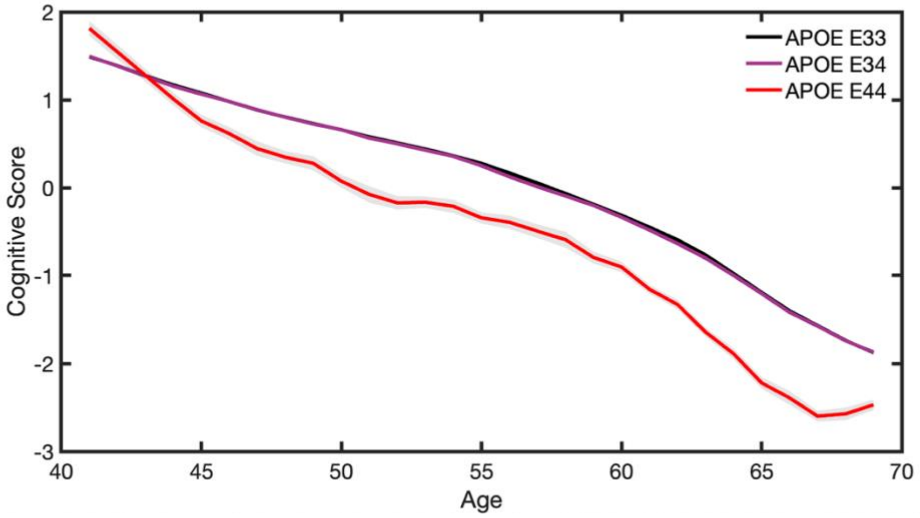

Supplemental Figure 2: Dose-dependent effect of *APOE ε4 on cognitive performance*

Comparison of cognitive composite scores among *APOE* ε33 (black), ε34 (purple) and ε44 (red) genotypes revealed genotype and age-dependent effects on cognition across both sexes combined. Cognitive scores are represented using a three-year moving average with individual's ages rounded to the nearest integer. Shaded area represents standard error of the mean (SEM) for each age group.

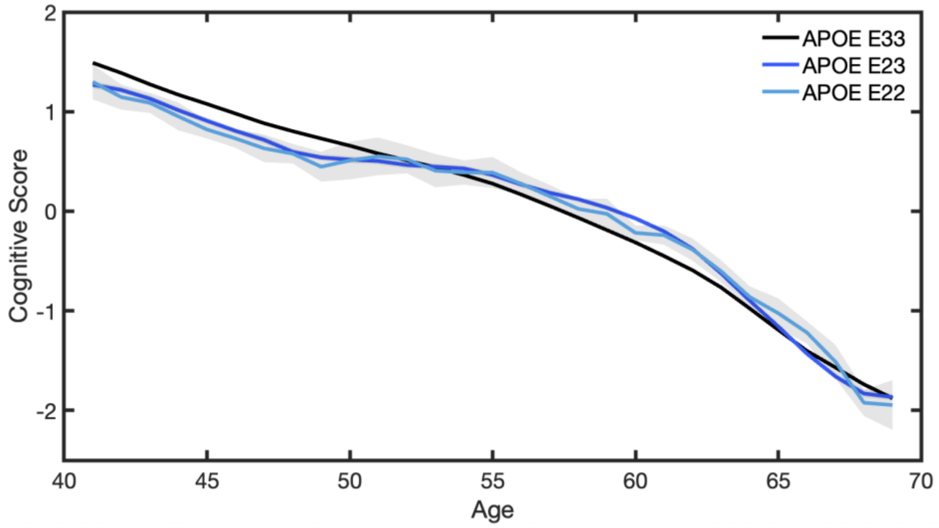

Supplemental Figure 3: Dose-dependent effect of *APOE ε2 on cognitive performance*

Comparison of cognitive composite scores among *APOE* ε33 (black), ε23 (dark blue) and ε22 (light blue) genotypes revealed genotype and age-dependent effects on cognition across both sexes combined. Cognitive scores are represented using a three-year moving average with individual's ages rounded to the nearest integer. Shaded area represents standard error of the mean (SEM) for each age group.

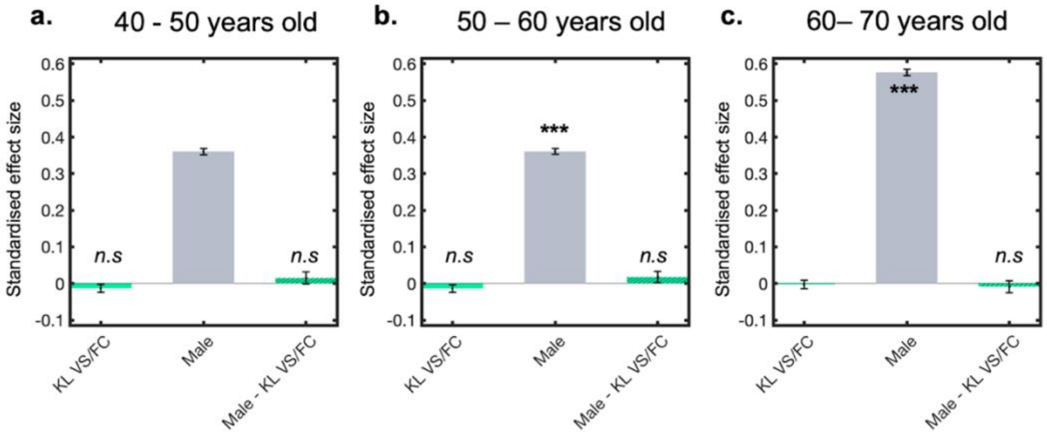

Supplemental Figure 4: Age-dependent effect of *KL-VS and sex on cognitive performance*

Panel a-c: Coefficient values for the main effects of Klotho and sex, and their interaction, analysed through sub-sampling across three age groups: 40-50, 50-60, and 60-70 years. Error bars represent the SEM. Significance levels are indicated as *p < 0.05, **p < 0.01, ***p < 0.001.

Age: 40-50

Variable	Estimate	SE	tStat	pValue
(Intercept)	0.864	0.005	166.840	< 0.001 ***
APOE E44	0.115	0.030	3.782	< 0.001 ***
APOE E22/23	-0.073	0.014	-5.137	< 0.001 ***
sex	0.413	0.008	53.211	< 0.001 ***
APOE E44:sex	-0.698	0.046	-15.265	< 0.001 ***
APOE E22/23:sex	-0.245	0.021	-11.474	< 0.001 ***

Age: 50-60

Variable	Estimate	SE	tStat	pValue
(Intercept)	-0.003	0.005	-0.625	0.532
APOE E44	-0.850	0.030	-28.461	< 0.001 ***
APOE E22/23	0.100	0.014	7.346	< 0.001 ***
sex	0.352	0.007	47.000	< 0.001 ***
APOE E44:sex	0.518	0.045	11.536	< 0.001 ***
APOE E22/23:sex	0.006	0.020	0.279	0.781

Age: 60-70

Variable	Estimate	SE	tStat	pValue
(Intercept)	-1.357	0.005	-246.880	< 0.001 ***
APOE E44	-0.755	0.033	-22.611	< 0.001 ***
APOE E22/23	0.091	0.015	6.034	< 0.001 ***
sex	0.581	0.008	72.759	< 0.001 ***
APOE E44:sex	-0.206	0.048	-4.315	< 0.001 ***
APOE E22/23:sex	-0.005	0.022	-0.226	0.821

Supplemental Table 1: Statistical Analysis of *APOE* and Sex Effects Across Age Ranges

Effects of *APOE* and sex across different age ranges, detailing each predictor and interaction. Significant results are highlighted in bold. The coefficients and standard errors (SE) are illustrated in Figure 1, Panels i and j.

	**Total N (Imaging)**	**Average education**	**Mean Bilateral hippocampal volume (mm^3) (SD)**
**APOE ε3/3 & ε3/4**	271199 (24967)	High 79.80%, Intermediate 6.26%, Low 13.93%	8567.12 (645.26)
**APOE ε4/4**	7854 (678)	High 81.85%, Intermediate 4.34%, Low 13.77%	8469.45 (712.58)
**APOEε2/3 & ε2/2**	41808 (3865)	High 79.70%, Intermediate 6.19%, Low 14.1%	8545.14 (645.26)
**KL FC/FC**	226303 (20723)	High 79.91%, Intermediate 6.25%, Low 13.85%	8565.89 (650.57)
**KL VS/FC**	86221 (7986)	High 79.62%, Intermediate 6.15%, Low 14.23%	8555.22 (652.48)

Supplemental Table 2: Demographics table representing APOE, education level and average hippocampal volume of UK Biobank cohort

Demographic information on participants, including APOE genotype (ε2, ε3, ε4 alleles), education level (years of formal education grouped by high = college or university degree, vocational degree. Intermediate = A/AS levels or equivalent. Low = O levels/GCSEs or equivalent), and hippocampal volumes (measured in mm^3 or standardised units).
